# Solitary Osteochondroma of the Thoracic Spine with Compressive Myelopathy; A Rare Presentation

**DOI:** 10.5812/iranjradiol.12015

**Published:** 2013-05-20

**Authors:** Payam Mehrian, Mohammad Ali Karimi, Shahram Kahkuee, Mehrdad Bakhshayeshkaram, Reza Ghasemikhah

**Affiliations:** 1 National Research Institute of Tuberculosis and Lung Disease, Shahid Beheshti University of Medical Sciences, Tehran, Iran; 2 Department of Radiology, Shahid Beheshti University of medical sciences, Tehran, Iran; 3 Department of Parasitology & Mycology, Arak University of medical sciences, Arak, Iran

**Keywords:** Osteochondroma, Spine, Spinal Cord Compression

## Abstract

A 19-year-old man presented with a 5-year history of back pain radiating to the lower extremities and paresthesis of the toes during the last year. Plain X-ray revealed a large cauliflower shaped exophytic mass at the level of T8, T9 and T10 vertebrae. Computed tomography (CT) and magnetic resonance imaging (MRI) showed an abnormal bony mass arising from the posterior arch of T9 with protrusion to the spinal canal and marked cord compression. The cortex and medulla of the lesion had continuity with those of the T9 vertebra. Surgical en bloc resection was performed and the patient’s symptoms resolved. The histopathologic diagnosis was osteochondroma. In patients with symptoms of myelopathy, in addition to more common etiologies, one should also be aware of rare entities such as osteochondroma.

## 1. Introduction

Osteochondromas, also known as exostoses, are the most common primary bone tumors comprising more than one third of all benign bone tumors (1). They can be solitary (90% of cases) or multiple in the form of hereditary multiple exostosis (HME) (10% of cases) (2). Osteochondromas are located frequently in the long bones and rarely involve the spine, representing only 2.6% of benign tumors of the spine (1-3). The incidence of spinal involvement (3% of cases) and neurological complications in HME is higher than solitary osteochondromas (4-6). Solitary vertebral osteochondromas with spinal cord compression are extremely rare and only 51 cases have been published until 2007 (7). Here, we report an additional case of osteochondroma of the thoracic spine with spinal cord compression.

## 2. Case Presentation

A 19-year-old man presented to the neurology clinic of Valiasr hospital in Arak (Iran) with a 5-year history of back and lower extremity pain. During the last year, he also had numbness and paresthesia of the toes. There was no history of previous trauma or surgery and the past medical history was unremarkable. The neurologic exam confirmed bilateral spastic paraparesia with bilateral hyperactive deep tendon reflexes in both legs and a positive Babinski response.

**Figure 1.**
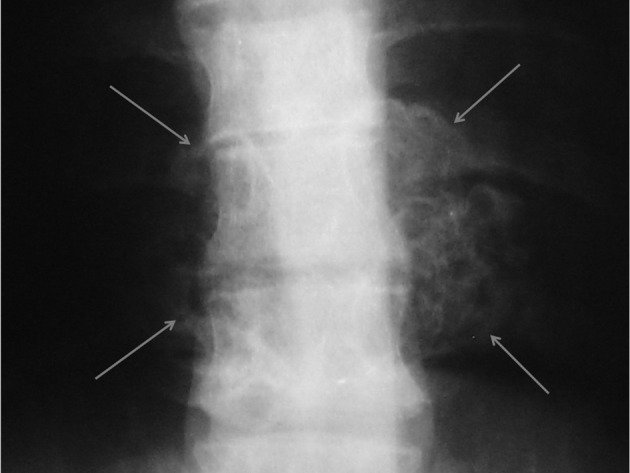
AP plain X-ray of the thoracic spine. A heterogeneous bony mass is seen in the lower thoracic spine.

Plain X-ray of the thoracic region revealed a large cauliflower shaped exophytic mass at the level of T8, T9 and T10 vertebrae (Figure 1). Computed tomography (CT) and magnetic resonance imaging (MRI) of the thoracolumbar region were performed to evaluate the nature and extent of the mass and its relationship with the spinal cord.

**Figure 2.**
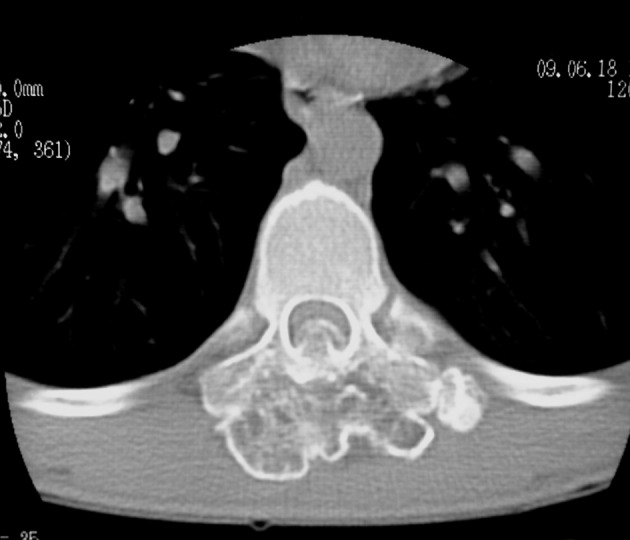
Bone window axial computed tomography scan at the level of T9 showing an abnormal bony mass arising from the posterior arch of T9 with extension to the spinal canal and compression of the spinal cord.

Bone window computed tomography scan showed an abnormal bony mass arising from the posterior arch of T9 with protrusion to the spinal canal and marked compression of the spinal cord (Figure 2). CT also demonstrated continuity of cortex and medulla of the lesion with those of the T9 vertebra, which is the most essential point for diagnosis of an exostosis. The spinous process and laminae of T9 vertebra were conglomerated with the mass.

**Figure 3.**
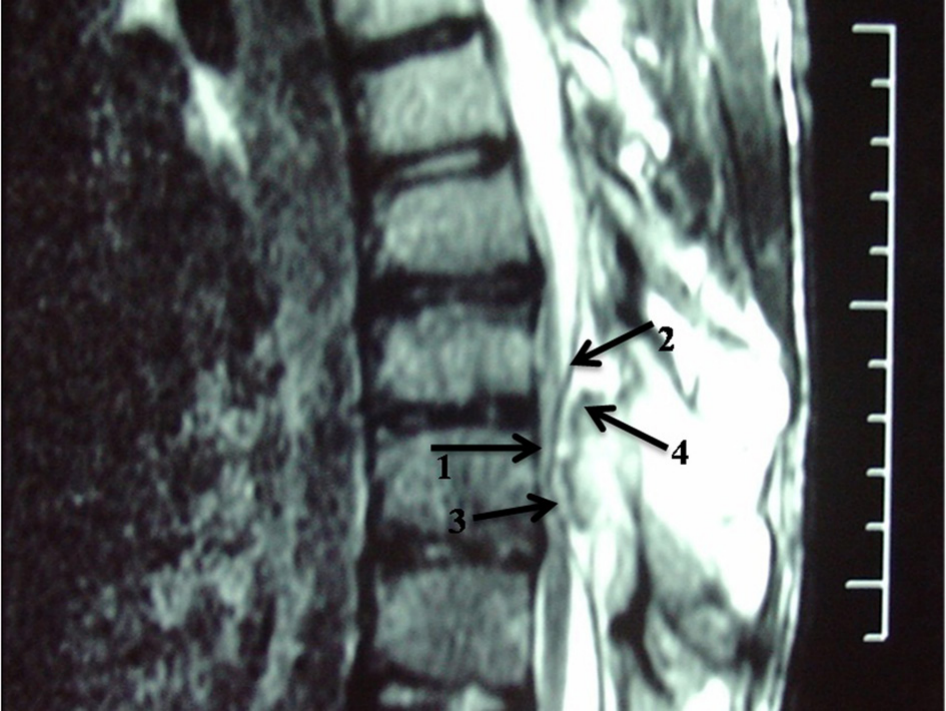
Sagittal T2-weighted MRI. Note the abnormal signal of the thinned and compressed cord (1), the displaced ligamentum flavum (2), the cartilage cap (3) and the cortical bone layer (4)

**Figure 4.**
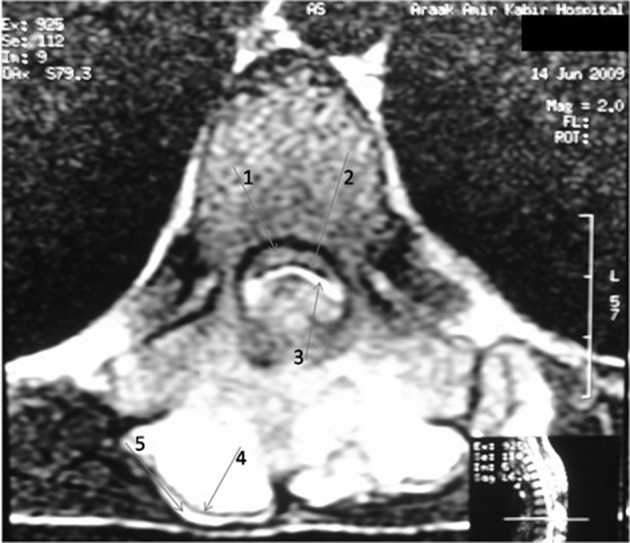
Axial T2-weighted MRI at the same level of Figure 3. Note the abnormal signal of the thinned and compressed cord (1), the displaced ligamentum flavum (2), the cartilage cap (3, 5) and the cortical bone layer (4)

Magnetic resonance imaging (MRI) confirmed presence of a bony mass, compressing the spinal cord. Cortical bone had low signal intensity in all pulse sequences, and the medullary component had an appearance of a yellow marrow. The cartilage cap was seen as a thin peripheral band (maximum thickness: 3mm) with intermediate to low signal intensity on T1-weighted images and high signal intensity on T2-weighted images (Figures 3 and 4).

At surgery, a predominantly bony mass originating from the lamina of T9 vertebra was removed. Histopathologic study showed a bony lesion with a cartilaginous cap indicating osteochondroma.

At the follow-up period, the patient’s symptoms completely resolved and neurologic functions gradually returned to normal.

## 3. Discussion

Osteochondromas are developmental lesions rather than true neoplasms (1). These lesions result from separation of a fragment of the epiphyseal growth plate cartilage, which subsequently herniates through the periosteal bone cuff that normally surrounds the growth plate (8). Trauma or genetic factors cause perichondral deficiency. A zone of metaplastic cartilage is produced and enlarged by enchondral ossification (9). They can be solitary (90% of cases) or multiple in the form of hereditary multiple exostosis (HME) (in 10% of cases), usually with autosomal dominant inheritance (2). The solitary and multiple forms are detected in males more frequently than in females with a ratio of 1.8:1 (multiple) to 3:1 (solitary) (10). Osteochondroma is a disease of the growing bone. Therefore, it often presents in young patients and its growth usually arrests after puberty with closure of the epiphysis (11, 12). Most of them are asymptomatic and are found incidentally. Symptomatic lesions usually occur in younger patients, with 75%-80% of such cases being discovered before the age of 20 years (5, 8). Patients with HME have at least two lesions that typically involve the proximal part of the humerus and the distal and proximal portions of the femur, tibia and fibula, but they may arise anywhere in the skeleton (1). There are often associated defects of bone modeling and bony deformities. HME leads to abnormalities such as palpable bony masses and limb shortening in the first or second decade of life (1, 8). The multiplicity of lesions and associated deformities lead to early radiologic evaluation and diagnosis of HME, in contrast to the relatively later diagnosis of solitary osteochondromas (13).

The HME cases are more likely complicated. Complications of exostoses include adjacent vascular and neural injury, fracture, osseous deformity, bursal formation and malignant degeneration (1). Exostoses may also be radiation-induced (9). Malignant transformation to chondrosarcoma is the most serious complication with an incidence of 1-5% for the solitary type and 10-25% for HME (1, 9, 14). Incomplete excision can lead to recurrence, so complete "en bloc" resection in the first operation is recommended and often curative (1, 15).

Spinal involvement is more common in HME. In this condition, thoracic and lumbar vertebrae are more commonly affected, while the solitary type mostly affects the cervical spine particularly the atlantoaxial region (1). Sacral involvement is rare (9). High prevalence in the cervical spine may be attributed to greater mobility and stress in this region. Lamina is the origin of most spinal osteochondromas. Extension into the spinal cord and cord compression, as occurred in our patient (Figures 2, 3 and 4), is a rare event. This usually occurs in the setting of HME (1). Our case is unusual in that a solitary vertebral exostosis has caused spinal cord compression. The most important clue in the diagnosis of osteochondroma is continuity between the cortex and medulla of the lesion and those of the underlying bone (2). In fact, this feature is pathognomonic for osteochondroma (1). As in our patient (Figure 3), this can be well-demonstrated by CT (6).

Diagnosis of spinal exostosis may be difficult on plain radiography owing to the complex anatomy (1, 9). X-ray is normal in 15% of the patients (6). However, in our patient this lesion manifested as a large cauliflower-like exophytic mass at the level of T8, T9 and T10 vertebrae (Figure 1). Lesions that protrude dorsally from the posterior vertebral elements (lamina or spinous process) are typically large and manifest at an earlier age with cosmetic deformity and a palpable mass, but lack neurologic symptoms. In contradistinction, osteochondromas that extend into the spinal canal are often small, but are associated with neurologic symptoms (1). Our case had both dorsal and ventral protrusions, causing a spinal mass with neurologic symptoms.

Malignant degeneration is best assessed by measuring the maximal cartilage cap thickness and MRI is more accurate than CT in this regard (16). It is supposed that thin caps (less than 3 cm) and calcified caps are benign and thick caps (more than 3 cm) are malignant (9, 16). In the presented case, maximal cartilage cap thickness was 3 mm (Figure 4), and pathology revealed no evidence of malignant transformation.

MRI often shows yellow marrow signal in the central aspect of the lesion. The cartilage cap is often thin and has low to intermediate signal on T1-weighted images and high signal on T2-weighted images (Figures 3 and 4).

Fat signal intensity within the medullary component of the spinal osteochondroma in MR imaging can occasionally be mistaken for a lipomatous neoplasm, particularly in small lesions projecting into the spinal canal (1). Thin-section CT is the modality of choice to demonstrate the diagnostic appearance of marrow and cortical continuity in the rib head, skull base, and spinal osteochondromas that often have a very narrow stalk of attachment. Nevertheless, MR imaging is usually superior to CT in evaluating the relationship of the osteochondroma to the surrounding structures for presurgical assessment (1).

Our case highlights the value of MRI for early diagnosis of spinal osteochondroma. Obviously, early diagnosis and therefore early treatment would prevent future permanent neurologic deficits. In cases of spinal cord compression, in addition to more common causes like discopathy, trauma and metastasis, one should be also aware of rare entities such as osteochondroma and this is particularly important in patients with HME. Whether MRI of the spine should be used as a screening test in HME can be a subject for future studies.

## References

[1] Murphey MD, Choi JJ, Kransdorf MJ, Flemming DJ, Gannon FH (2000). Imaging of osteochondroma: variants and complications with radiologic-pathologic correlation.. Radiographics..

[2] Edelman RR, Hesselink JR, Zlatkin MB (2006). Clinical magnetic resonance imaging..

[3] Hassankhani EG (2009). Solitary lower lumbar osteochondroma (spinous process of L3 involvement): a case report.. Cases J..

[4] Khosla A, Martin DS, Awwad EE (1999). The solitary intraspinal vertebral osteochondroma. An unusual cause of compressive myelopathy: features and literature review.. Spine (Phila Pa 1976)..

[5] Scarborough MT, Moreau G (1996). Benign cartilage tumors.. Orthop Clin North Am..

[6] Murphey MD, Andrews CL, Flemming DJ, Temple HT, Smith WS, Smirniotopoulos JG (1996). From the archives of the AFIP. Primary tumors of the spine: radiologic pathologic correlation.. Radiographics..

[7] Song KJ, Lee KB (2007). Solitary osteochondroma of the thoracic spine causing myelopathy.. Eur J Pediatr Surg..

[8] Resnick D, Kyriakos M, Greenway GD (1995). Osteochondroma. In: Resnick D, ed. Diagnosis of bone and joint disorders..

[9] Quirini GE, Meyer JR, Herman M, Russell EJ (1996). Osteochondroma of the thoracic spine: an unusual cause of spinal cord compression.. AJNR Am J Neuroradiol..

[10] Albrecht S, Crutchfield JS, SeGall GK (1992). On spinal osteochondromas.. J Neurosurg..

[11] Chazono M, Masui F, Kawaguchi Y, Hazama H, Ueda J, Saito S (2009). Dumbbell-shaped osteochondroma of the fifth rib causing spinal cord compression.. J Orthop Sci..

[12] Shim JH, Park CK, Shin SH, Jeong HS, Hwang JH (2012). Solitary osteochondroma of the twelfth rib with intraspinal extension and cord compression in a middle-aged patient.. BMC Musculoskelet Disord..

[13] Carroll KL, Yandow SM, Ward K, Carey JC (1999). Clinical correlation to genetic variations of hereditary multiple exostosis.. J Pediatr Orthop..

[14] Bell RS (1999). Musculoskeletal images. Malignant transformation in familial osteochondromatosis?. Can J Surg..

[15] Lotfinia I, Vahedi P, Tubbs RS, Ghavame M, Meshkini A (2010). Neurological manifestations, imaging characteristics, and surgical outcome of intraspinal osteochondroma.. J Neurosurg Spine..

[16] Woertler K, Lindner N, Gosheger G, Brinkschmidt C, Heindel W (2000). Osteochondroma: MR imaging of tumor-related complications.. Eur Radiol..

